# Atypical Porcine Pestiviruses: Relationships and Conserved Structural Features

**DOI:** 10.3390/v13050760

**Published:** 2021-04-26

**Authors:** Christiane Riedel, Hazel Aitkenhead, Kamel El Omari, Till Rümenapf

**Affiliations:** 1 Institute of Virology, Department of Pathobiology, University of Veterinary Medicine Vienna, 1210 Vienna, Austria; till.ruemenapf@vetmeduni.ac.at; 2 Diamond Light Source, Harwell Science and Innovation Campus, Didcot OX11 0DE, UK; hazel.aitkenhead@diamond.ac.uk (H.A.); kamel.el-omari@diamond.ac.uk (K.E.O.); 3 Rutherford Appleton Laboratory, Research Complex at Harwell, Didcot OX11 0FA, UK

**Keywords:** pestivirus, atypical porcine pestivirus, phylogeny, structural relationsship

## Abstract

For two decades, the genus pestivirus has been expanding and the host range now extends to rodents, bats and marine mammals. In this review, we focus on one of the most diverse pestiviruses, atypical porcine pestivirus or pestivirus K, comparing its special traits to what is already known at the structural and functional level from other pestiviruses.

## 1. Introduction

**Figure 1:**
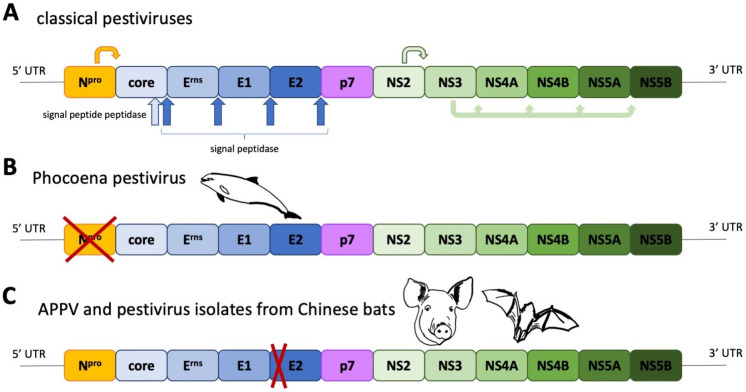
(**A**) Pestivirus genome organisation, polyprotein processing and special traits of viruses isolated from harbour porpoises (**B**) as well as swine and bats (**C**). The structural proteins are coloured in shades of blue. NS = non-structural protein.

Pestiviruses, as enveloped, single-stranded, positive sense RNA viruses, are members of the *Flaviviridae*. They can be distinguished from other members of this virus family by the presence of three surface glycoproteins, E^rns^, E1 and E2, and an N-terminal autoprotease, Npro. The genome organisation and polyprotein processing are depicted in Figure 1A. For several decades, the classical pestiviruses, represented by economically important pathogens of cloven-hoofed animals, such as Bovine viral diarrhoea Virus (BVDV) and Classical swine fever virus (CSFV), constituted all known species. Starting with the discovery of Bungowannah virus in Australia 2007 [1], this drastically changed, as even more divergent isolates were identified in bats [2,3], rodents [3,4], harbour porpoises [5] and pigs [6,7,8]. This also overthrew the theorem that pestiviruses are solely infecting cloven-hoofed animals. In the following, we will discuss the phylogeny of atypical porcine pestiviruses and compare the characteristics of these newly discovered porcine viruses.

## 2. Phylogeny and Geographic Distribution of Atypical Porcine Pestiviruses

**Figure 2:**
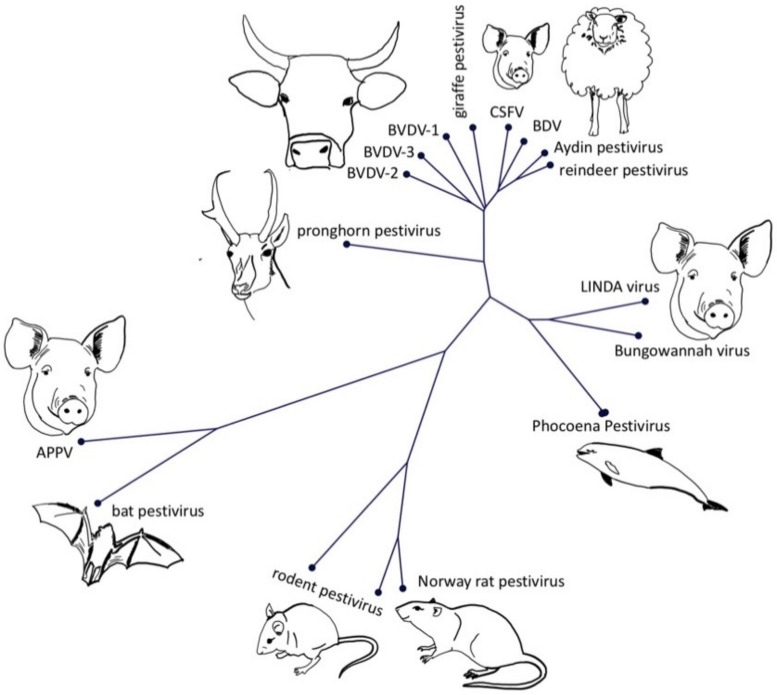
Neighbour joining tree (1000 replicates) of the polyprotein of representatives of different pestivirus species and isolates. GenBank accession numbers of the isolates are: APPV AQM73625, Aydin-like pestivirus YP_006860588.1, bat pestivirus AYV99177.1, BDV AAC16444.1, Bungowannah virus YP_008992092.1, BVDV-1 AYA62524.1, BVDV-2 AMO65207.1, BVDV-3 AGO04420.1, CSFV CAA65386.1, LINDA virus YP_009407716.1, Norway rat pestivirus YP_009109567.1, pestivirus giraffe NP_620053.1, pestivirus isolate reindeer AAF02524.2, phocoena pestivirus QFQ60724.1, pronghorn antelope pestivirus YP_009026415.1, rodent pestivirus ATP66856.1 and ATP66855.1. The phylogenetic tree was generated with CLC Sequence Viewer and the multiple sequence alignment with ClustalW.

The term atypical porcine pestivirus has been criticized in the literature due to its ambiguity, caused by the discovery of different, highly divergent pestivirus isolates in domestic pigs. This has led to recent changes in the nomenclature of pestiviruses, assigning letters in an alphabetical order to all recognized pestivirus species [9]. The first atypical porcine pestivirus to be discovered was the Bungowannah virus (Pestivirus F), which has been discovered on an Australian pig farm and has been restricted to this property and a neighbouring farm [1]. The next atypical porcine pestivirus (APPV, Pestivirus K) was discovered during a PRRSV metagenomic sequencing project [8] and was quickly associated with congenital tremor [7,10,11]. Just two years later, another atypical porcine pestivirus—lateral shaking inducing agent (LINDA) virus—was reported in piglets presenting with congenital tremor originating from a single Austrian farm [6]. Although LINDA virus and APPV are both associated with congenital tremor, they have no close phylogenetic relationship (Figure 2). Bungowannah virus and LINDA virus are closest related to recently discovered pestiviruses from harbour porpoises [5], even though these porpoise pestiviruses do not possess an Npro coding region (Figure 1B). The closest relatives of APPV are pestiviruses originating from Chinese bats (*Rhinolophus affinis*, *Scotophilus kuhlii*) [2,3], whilst pestiviruses discovered in rodents in China (*Apodemus draco*, *Apodemus peninsulae*, *Niviventer excelsior*, *Niviventer niviventer*) [12] and the USA (*Rattus norvegicus*) [4] seem to form a phylogenetically distinct clade. Both APPV and bat isolates have a deletion of nearly the complete two N-terminal structural domains of the E2 glycoprotein (Figure S1).

**Figure 3:**
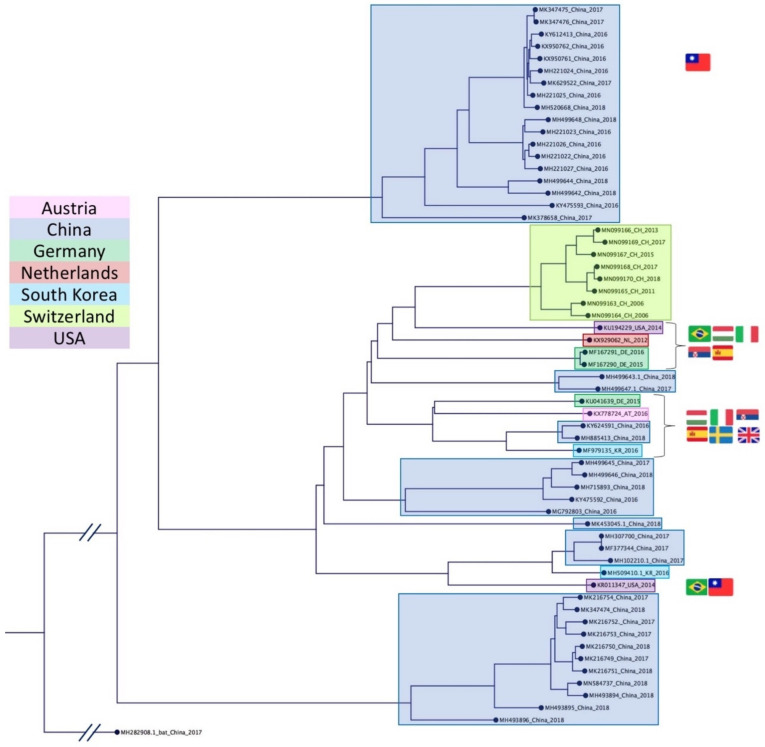
Neighbour Joining tree of full ORF coding sequences of APPV isolates available from GenBank. The tree was generated from 1000 replicates and a bat pestivirus isolate from China has been used as an outgroup (MH282908). GenBank accession numbers as well as the year of isolation are given for each isolate in the tree. Isolates are coloured according to geographic origin. Flags indicate the closest related full ORF sequence in case no full ORF coding sequences are available from a country where APPV has been reported. The phylogenetic tree was generated with CLC Sequence Viewer and the multiple sequence alignment with ClustalW.

APPV has been isolated in all continents, apart from Africa and Australia (Figure 3). In Europe, APPV in domestic pigs has been documented in Austria [13], Germany [11,14,15], Hungary [16], Italy [14,17], Serbia [14], Spain [18], Sweden [19,20], Switzerland [14,21] and the United Kingdom [14,22] and in wild boar in Germany [23] and Spain [24]. In the Americas, reports of APPV infection exist from Brazil [25,26], Canada [27] and the USA [7,8,28]. Asian APPV strains are available from China [29,30,31,32,33,34,35,36,37,38], Taiwan [14] and South Korea [39]. The highest sequence diversity to date has been detected in China. Based on the divergent nature of APPV genomes, the assignment of three [38] or four [37] genotypes for the full ORF coding region has been proposed recently. Of these proposed genotypes, two have only been identified in China. Partial sequences mainly employed for phylogenetic analysis are located in the NS2-3 or NS5B coding regions, due to their high degree of sequence conservation. Whether the utilisation of the E2 coding regions—as practiced for example for CSFV [40]—is advantageous over the aforementioned regions, will have to be shown in the future. Long term monitoring of an APPV infected herd suggests that immunological pressure on the glycoproteins is low, as no substantial amino acid exchanges could be observed [41].

Serological assays have also been implemented to assess the exposure of pig populations to APPV on a larger scale but are not yet commonly used for the determination of APPV seroprevalence. A competitive ELISA employing the APPV NS3 helicase domain and a cross-reactive mouse monoclonal antibody generated against CSFV and BVDV NS3 helicase has been developed by Schwarz et al. [13]. E^rns^, recombinantly expressed in and secreted by *Leishmania tarentolae*, was used by Postel et al. [42] as the antigen in an indirect ELISA setup, whilst two E^rns^ peptides expressed in *E. coli* were employed by Hause et al. [8]. Based on the *Leishmania tarentolae* expression system, an indirect E2 ELISA has also been developed and its comparison with the neutralizing activity of detected antibody showed a proportional increase of antibody levels and neutralization, which could not be shown for antibodies directed against E^rns^ [43]. Therefore, it seems likely that APPV E2 is the major target for neutralizing antibodies as observed for other pestiviruses. Reported seroprevalences of antibodies against APPV range between 47–86% in domestic pigs, depending on the geographic origin [14]. APPV genomes could be detected in 2.3–17.5% of the tested animals in the same study depending on the country of origin. In Spain, 13.9% of samples tested positive for APPV genome [18]. For Germany, Beer et al. [15] reported a genome prevalence of 9% in slaughter pigs and 20% in sera derived from farms whilst Kaufmann et al. [21] detected a higher seroprevalence (13%) in slaughter pigs than in breeding pigs (<1%) in Switzerland. Semen samples and preputial swabs of commercial boar farms in the USA tested positive in 15.8% [28] and this number is in good accordance with the 16.4% genome detection rate reported by Chen et al. [44] for the USA. Interestingly, a seroprevalence of 52% and a genome detection rate of 19% was determined for wild boar samples from Germany [23], indicating a wide spread of the pathogen also in wild populations. The earliest positively tested samples originate from Spain (1997) [18] and Switzerland (1986) [21].

## 3. Special Traits of Atypical Porcine Pestiviruses

APPV possesses the same genomic traits as all other pestiviruses (apart from phocoena pestivirus) regarding the presence of coding regions of all known pestiviral proteins.

The sequence conservation between different proteins of the polyprotein is varying strongly. Npro, p7, NS2 and NS5A are least conserved, with less than 5% of amino acids being identical for all known pestiviruses. Core, E1, E2 and NS4A show an intermediate degree of amino acid identity, varying between 9 and 15%. E^rns^ (23%), NS3 (29%), NS4B (20%) and NS5B (22%) have the highest degree of amino acid identity conservation.

**Figure 4:**
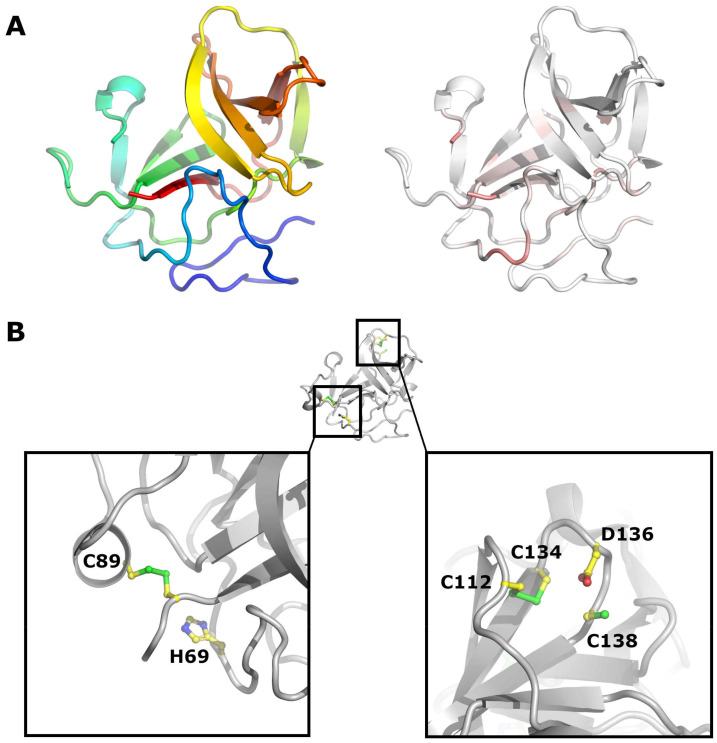
Crystal structure of CSFV Npro. (**A**) Cartoon representation of Npro (PDB code 4H9J). On the left, Npro is coloured from the N terminus (blue) to the C terminus (red). On the right, Npro is coloured according to sequence conservation from white (non-conserved) to red (conserved). (**B**) Location of important residues in Npro, H69 and C89 (left, APPV numbering) and the TRASH motif (right) are drawn as sticks. Figure 4, Figure 5, Figure 6, Figure 7 and Figure 8 were prepared with PyMOL (The PyMOL Molecular Graphics System, Schrödinger, LLC., New York, NY, USA).

The low degree of conservation in the Npro coding region is surprising (Figure 4A and Figure S2), as Npro plays a pivotal role in innate immune evasion by targeting a central transcription factor of the Interferon system—IRF3—for proteasomal degradation [45,46,47,48], thereby circumventing the development of an antiviral status of the host cells. Only the residues pivotal for the Npro autoprotease domain are conserved in all pestiviruses, for example, His69 and Cys89 (all amino acid positions are given as the residue number on the APPV polyprotein, if not indicated otherwise), as well as Cys180, which is just N-terminal of the Core N-terminus (Figure 4B). The TRASH motif (Cys112-Cys134-Asp136-Cys138 of CSFV, Figure 4B), as potential coordinator of a zinc atom, is indispensable for Npro’s IRF-3 antagonistic activity [49] and absent in APPV. Yet, recent data indicate that APPV possesses IRF-3 antagonistic activity, but the mechanism of action will still need to be determined [50].

The amino acid identity of all pestiviral core proteins—which is important for virus assembly, an intrinsically disordered protein and harbours an RNA chaperone activity [51,52]—is 9%. APPV core protein is also characterised by an estimated isoelectric point of 10, which is similar to what has been reported for other pestiviral core proteins. This highlights the importance of positively charged residues for its functionality, which is likely pivotal to mediate binding of nucleotides. One functionally important motif seems to be Lys271-X-Lys273-X-X-Trp276 just N-terminal of the signal peptide peptidase cleavage site, which is conserved in all known pestiviruses.

**Figure 5:**
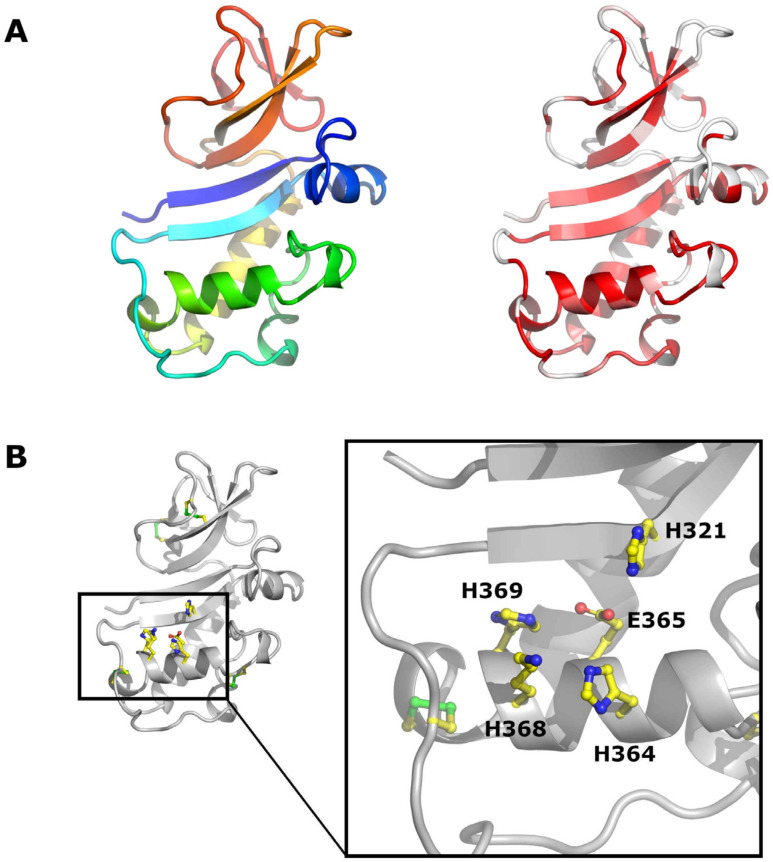
Crystal structure of E^rns^. (**A**) Cartoon representation of E^rns^ (PDB code 4DWC). On the left, E^rns^ is coloured from the N terminus (blue) to the C terminus (red). On the right, E^rns^ is coloured according to sequence conservation from white (non-conserved) to red (conserved). (**B**) Disulfide bridges and residues of the active site are shown as sticks.

E^rns^, like Npro, is another protein unique to pestiviruses (Figure 5A). Unlike Npro, it has the second highest degree of amino acid conservation among all pestiviral proteins (Figure 5A right and Figure S3), suggesting an important role for the pestiviral life cycle and a low tolerance to modifications. E^rns^ possesses an RNase activity, and its RNase domain is within the structural family of T2 RNases, that are mostly found in fungi and plants [53,54,55]. The active site consists of His321, His364, Glu365, Lys368, and His369 (Figure 5B). All cysteine residues involved in the formation of disulphide bridges—either intramolecular or during homodimer formation—are conserved among all pestiviruses (Figure S3). Only the N-glycosylation site at position 355 is present in all pestiviruses. E^rns^ is important for the generation of infectious particles [56,57], apart from Bungowannah virus [58], as an attachment factor due to an interaction with cell surface glycosaminoglycans [59] and as an antagonist of the innate immune system [57,60,61,62,63]. However, the proposed heparan sulphate interacting domain [64] is not conserved in pestivirus species discovered after 2000. In APPV, there is also no evidence of the presence of a cellular retention signal consisting of residues Leu183, Ile109 and Leu208 of CSFV E^rns^ [65].

E1 is a structural protein usually present as a heterodimer in complex with E2 in the virus particle [66]. This heterodimer is essential for infectivity [67,68] and heterodimer formation is thought to occur via interactions between the C-terminal transmembrane domains of E1 and E2. Lys679 and Lys682, as two key residues mediating this interaction [68], are conserved as basic amino acids in all pestiviruses. In contrast, Cys171 in the E1 of BVDV, which has been predicted to form a disulphide bridge with Cys296 [69], is not well conserved. Based on structural analysis of BVDV E2 [70,71], E1 has been proposed as the protein carrying a yet unknown fusion domain. Studies on CSFV however have proposed the location of a fusion domain within E2. Three Cysteine residues within E1 are conserved between all pestiviruses (506, 521, 628), indicating an important role in structure stabilization and potentially also heterodimer formation.

**Figure 6:**
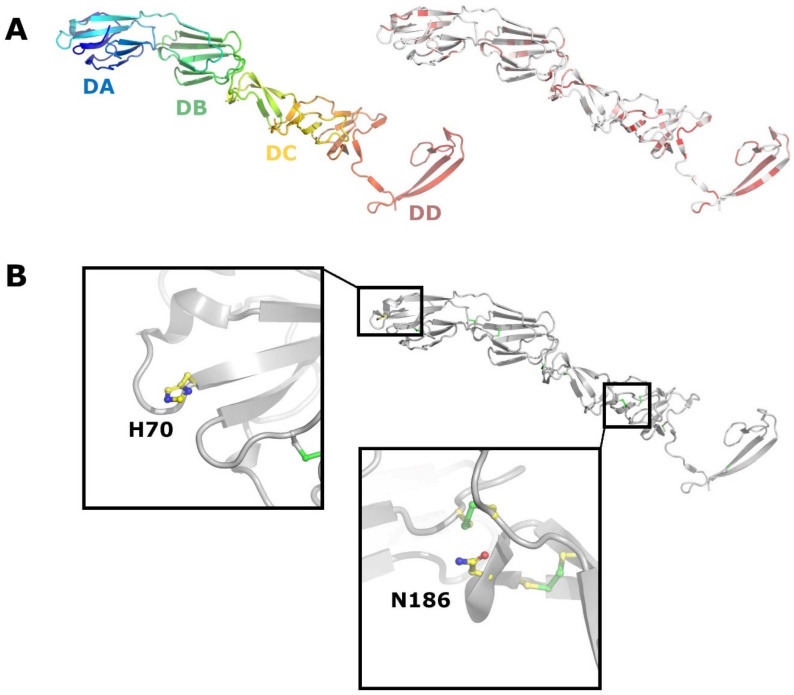
Crystal structure of BVDV E2. (**A**) Cartoon representation of E2 (PDB code 2YQ2). On the left, E2 is coloured from the N terminus (blue) to the C terminus (red). On the right, E2 is coloured according to sequence conservation from white (non-conserved) to red (conserved). (**B**) Residues H70 and N186 from the DA and DD domains respectively, are shown as sticks.

E2 is a major antigen and target for neutralizing antibodies. It is recognized as the receptor binding protein for both BVDV and CSFV. Structural studies on BVDV E2 report two membrane distal domains containing Ig-like domains (Figure 6A), and depending on the author, have been termed DA and DB [70] or I and II [71], respectively. The next domain DC is a highly extended structure consisting of loops and antiparallel β-strands and disulphide rich. One fairly conserved glycosylation site (Asn186 BVDV) is located in this domain (Figure 6B). This residue is however absent in APPV and pestivirus isolates from bats and rodents. Domain DD is involved in homodimer formation, including a domain swap, and the most conserved among pestiviruses. Domains DC and DD correspond to domain III proposed by Li et al. [71]. Upon a decrease in pH, domain DA becomes disordered, in a process that might expose a potential fusion region in E1 in analogy to Sindbis virus [72]. El Omari et al. [70] propose that the disordering of domain DA is coordinated by a His residue (H70 BVDV) that is conserved in all pestivirus isolates apart from those found in bats and rodents (Figure 6B). Intriguingly, the N-termini of APPV E2 and the E2 of pestiviruses isolated from bats are truncated by 130 aa, resulting in the loss of DA and most of DB (Figure S1). This might imply a different entry mechanism and a different functional assignment between E1 and E2. Of the eight disulphide bridges reported for BVDV E2 [70,71], only the eighth one is conserved in APPV at the sequence level (Figure S1). Five cysteine residues, involved in the formation of disulphide bridges 3–7, are not conserved in APPV, which could result in a totally different topology and stabilization of the protein. A C-terminal Arg residue in the transmembrane domain of E2 (BVDV R1047), which has been implicated as important for the E1-E2 heterodimer formation [68], is conserved as a basic residue in all pestiviruses, indicating a potentially genus wide functional importance (Figure S1). Bovine CD46 has been demonstrated to act as a receptor for BVDV [73,74] and its porcine counterpart has also been proposed as a receptor for CSFV [75]. However, recent results employing knock-out cell lines demonstrate that porcine CD46 is not a receptor for CSFV, but instead for APPV [76]. Given the differences between BVDV and APPV E2 already at the sequence level, this result is highly intriguing, as CD46′s structure is considered quite conserved across different species.

The p7 protein of CSFV has been reported to act as a viroporin [77,78,79,80], in analogy to HCV (reviewed in [81]). Its membrane topology is governed by two predicted alpha helices, that cross the ER membrane towards the cytoplasm and back. Although the overall sequence conservation of p7 at the level of single residues is very low, at least for APPV and pestiviruses from bats, all p7 sequences are high in hydrophobic residues and contain 2 basic residues at position 34 and 39 (for CSFV), which supposedly enclose the aa loop in contact with the cytoplasm.

NS2 is an autoprotease consisting of an N-terminal, hydrophobic domain and predicted to contain up to 7 transmembrane helices, and a C-terminal, cytoplasmic domain. Its protease activity is mediated by the catalytic triad of His1447, Glu1462 and Cys1512 [82] (with reference to the BVDV polyprotein). Sequence conservation between APPV and other pestiviruses, apart from the ones originating from bats, is rather low (10-15%) [8], and overall, only 4.2% of the amino acid residues are conserved amongst all pestiviruses. Two members of the catalytic triad, BVDV His1447 and Glu1462, can be identified in the NS2 of APPV (His1237 and Glu1253). The cysteine residue, however, is not present in APPV and bat pestiviruses, but might be compensated for by the cysteine residue at position 1280, rendering the amino acid stretch between Glu and Cys 23 residues shorter. The autoprotease function of NS2 relies on a cellular cofactor, Jiv [83,84], which is therefore essential for the replication of ncp pestiviruses [85]. Whether this dogma is also true for the rather diverse subset of pestiviruses including APPV and isolates of bats and rodents needs to be determined still.

**Figure 7:**
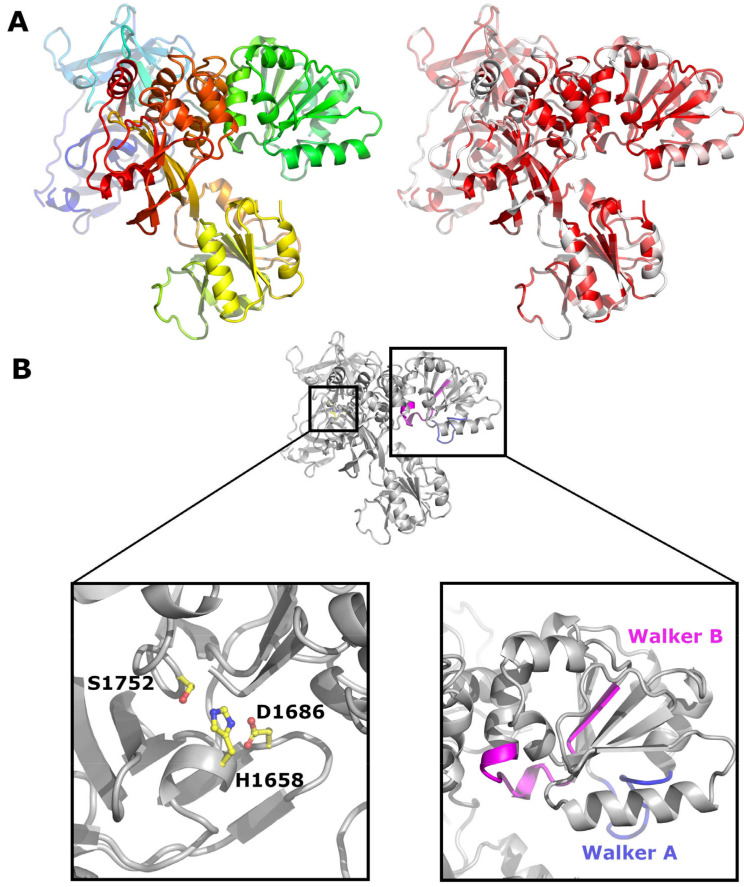
Crystal structure of CSFV NS3. (**A**) Cartoon representation of NS3 (PDB code 5WX1). On the left, NS3 is coloured from the N terminus (blue) to the C terminus (red). On the right, NS3 is coloured according to sequence conservation from white (non-conserved) to red (conserved). (**B**) On the left, residues forming the catalytic triad are shown as sticks; on the right, the Walker motifs A and B are coloured in blue and magenta respectively.

NS3 is a multifunctional protein which contains a chymotrypsin like serine protease as well as an ATPase and helicase domain [86,87,88] (Figure 7A). Its N-terminus, essential for protease function, is generated by NS2 cleavage. The protease activity is mediated by a catalytic triad consisting of His1658, Asp1686 and Ser1752 in BVDV [89,90] (Figure 7B). The residues of the protease domain are conserved in all pestivirus isolates and correspond to amino acids 1387, 1415 and 1481 of the APPV polyprotein (Figure S4). NS3 cleaves the polyprotein downstream of its own localisation between Leu in the P1′ position and Ser, Ala, or Asn in P1 [91,92,93] and also generates NS3 subsegments at cleavage sites containing either a Leu or Ile at position P1′ and a Met or Lys at P1 [94]. The cleavage sites corresponding to CSFV aa 1748/1749, generating an inactivated NS3 protease fragment, is conserved in all pestiviruses, whilst the cleavage site at CSFV aa position 1781/1782 is not conserved in pronghorn antelope pestiviruses, APPV and isolates from rodents and bats. With an amino acid identity of 29%, NS3 is the most conserved pestiviral protein. The Walker A and B motifs, required for helicase function, are also conserved in all pestiviruses and correspond to amino acids 1545-1553 and 1636-1648 in APPV (depicted in blue and magenta in Figure 7B).

NS4A is the essential cofactor of the NS3 protease and its C-terminal domain seems to be pivotal for cofactor activity [89]. At the sequence level, NS4A reveals an intermediate level of amino acid identity conservation of 15.2% between all pestiviruses.

NS4B is predicted as an integral membrane protein [95] and has certain sequence stretches resembling Walker A and B motifs [96]. Its overall amino acid conservation status in pestiviruses is 20.7%.

NS5A is an N-terminally membrane anchored protein and phosphorylated by cellular proteases [97]. It possesses a zinc coordination motif [98], which is conserved in all pestiviruses. However, the C-X22-C-X-C-X24 motif is modified to a C-X21-C-X-C-X23 motif in APPV and pestivirus isolates from bats. Interestingly, a fluorophore tag can be introduced into NS5A of BVDV replicons [99] and it might be worthwhile checking whether such a tool can be employed for real time tracking of replication of more divergent pestiviruses. CSFV NS5A is regulating viral RNA replication either by an interaction with the 3′ IRES or the modulation of the NS5B polymerase activity by direct protein-protein interaction [100,101,102,103,104]. CSFV NS5A residues Trp143, Val145, Pro227, Thr246, and Pro257 are important for replication and residues Lys399, Thr401, Glu406, and Leu413 are involved in IRES mediated translation and binding of NS5B. Both motifs are not conserved in APPV and pestivirus isolates from rodents, which is surprising as they are also conserved in HCV [100].

**Figure 8:**
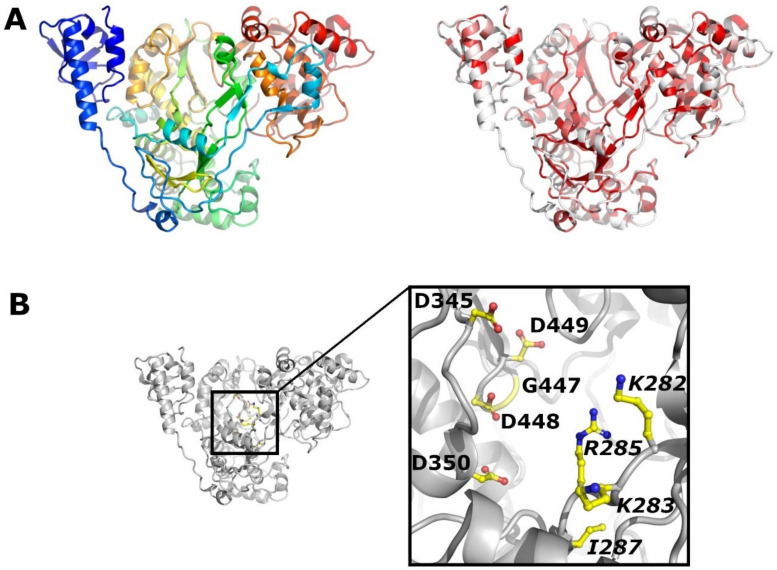
Crystal structure of NS5B. (**A**) Cartoon representation of NS5B (PDB code 5Y6R). On the left, NS3 is coloured from the N terminus (blue) to the C terminus (red). On the right, NS5B is coloured according to sequence conservation from white (non-conserved) to red (conserved). (**B**) The catalytic aspartate residues (D345 and S350) as well as the GDD motif (447–449) are shown as sticks. Important finger domain residues (K282, K283, R285 and I287) are labelled in italics.

NS5B is an RNA dependent RNA polymerase and relying on other non-structural proteins to function in the cellular environment. The structure of BVDV [105,106] and CSFV NS5B [107] has been determined by X-ray crystallography, revealing the classical palm, fingers and thumb domains of RNA polymerases (Figure 8A). The catalytic residues are conserved in all pestiviruses (CSFV Asp345 and Asp350, and the GDD motif at position 447–449) (Figure 8B), but an astonishingly low degree of sequence conservation is found in the N-terminal domain of NS5B, which is unique to the polymerase of pestiviruses and of functional importance (Figure S5). CSFV NS5B residues deemed important for the function of the finger domain, Lys282, Lys283, Arg285 and Ile287 [107] (Figure 8B), are present in all known pestiviruses and located in a well conserved sequence stretch.

## 4. Conclusions

The continuing discovery of additional, often diverse pestiviruses keeps on challenging accepted dogmas in pestivirus biology, like host restriction to cloven-hoofed animals and the conservation of (parts of) proteins considered essential for pestivirus replication, such as Npro or the E2 N-terminus. Albeit this increase in diversity, common functionalities can still be observed, even though the underlying molecular mechanism—such as Npro’s IFN antagonistic behaviour or CD46 binding—are likely differing. Further research will hopefully shine light on these interesting new aspects in pestivirus biology.

## Supplementary Materials

The following are available online at https://www.mdpi.com/article/10.3390/v13050760/s1, Figure S1: Protein sequence alignment of pestivirus E2 proteins, Figure S2: Protein sequence alignment of pestivirus Npro proteins, Figure S3: Protein sequence alignment of pestivirus E^rns^ proteins, Figure S4: Protein sequence alignment of pestivirus NS3 proteins, Figure S5: Protein sequence alignment of pestivirus NS5B proteins.
