# Supplementary material for: Atypical Porcine Pestiviruses: Relationships and Conserved Structural Features
Source: Viruses. 2021 Apr 26;13(5):760. doi: 10.3390/v13050760 (PMC8146772; doi:10.3390/v13050760)
Supplement: Supplementary file 1 [file viruses-13-00760-s001.zip › viruses-1194624-supplementary.pdf]

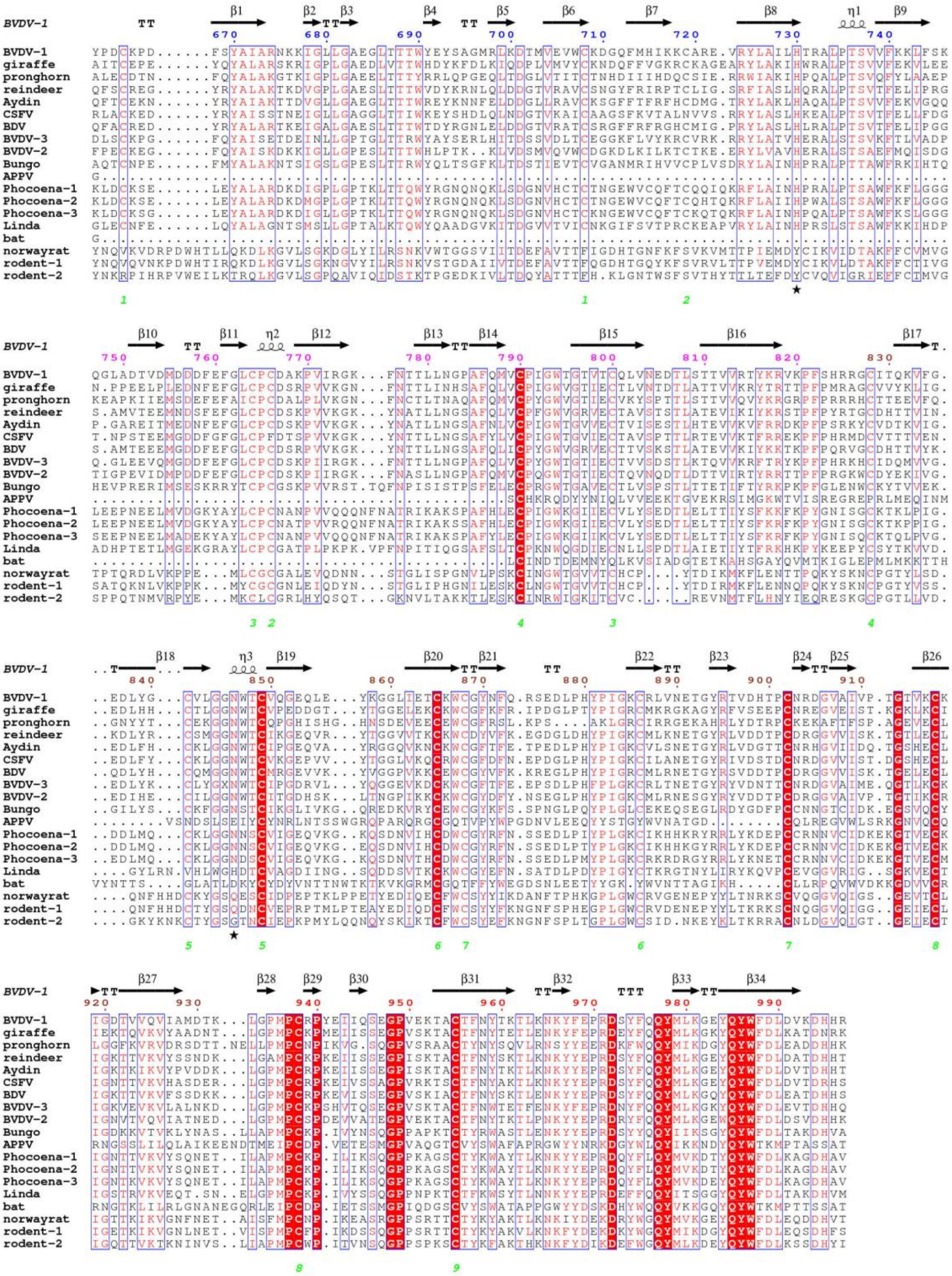

Figure S1. Protein sequence alignment of pestiviruses E2 proteins. Sequence alignment of BVDV1 E2 with E2 proteins from other pestiviruses. Conserved residues are drawn in red boxes, similar residues in red type. The secondary structure assignment for E2 is shown at the top. Cysteines involved in intra-chain disulfide bridges are marked by a green number and residues described in the text are marked by a star. Figure was prepared with ESPript (reference doi: 10.1093/nar/gku316).

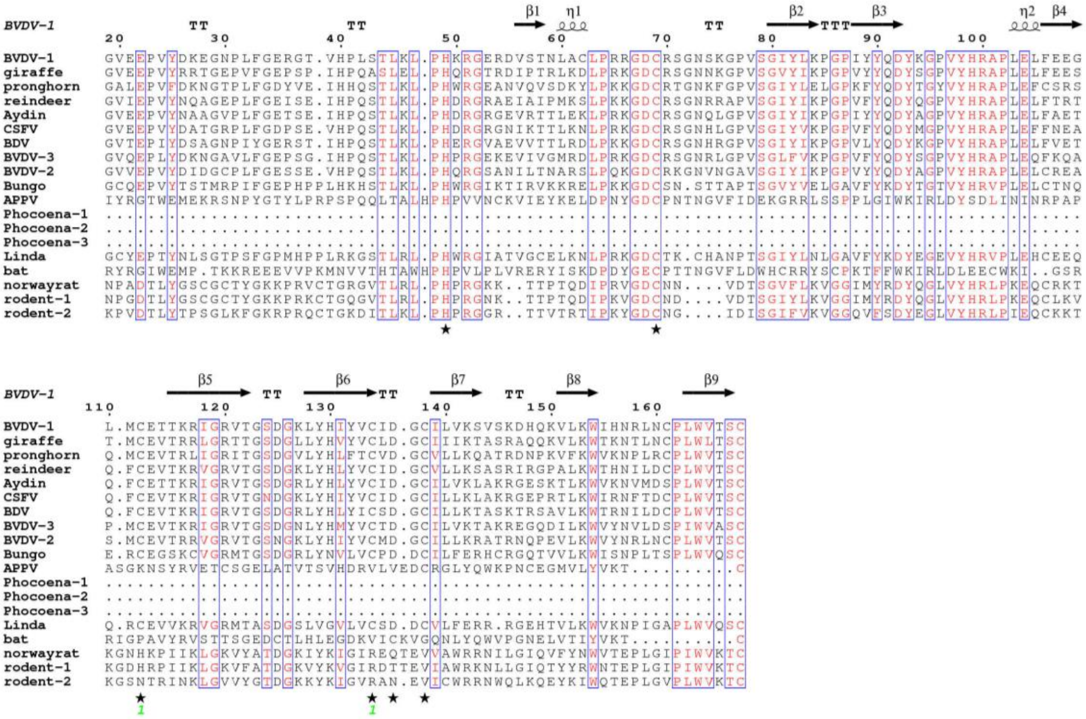

Figure S2. Protein sequence alignment of pestiviruses Npro proteins. Sequence alignment of BVDV1 Npro with Npro proteins from other pestiviruses. Conserved residues are drawn in red boxes, similar residues in red type. The secondary structure assignment for Npro is shown at the top. Cysteines involved in intra-chain disulfide bridges are marked by a green number and residues described in the text are marked by a star.

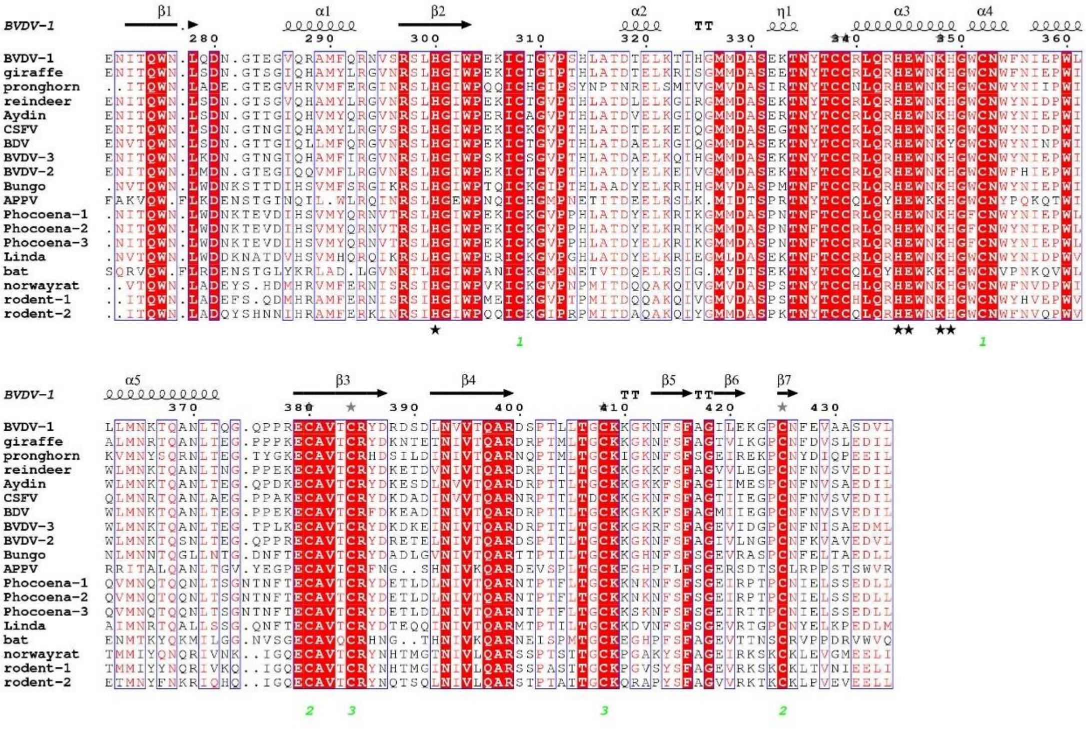

Figure S3. Protein sequence alignment of pestiviruses E^{rns} proteins. Sequence alignment of BVDV1 E^{rns} with E^{rns} proteins from other pestiviruses. Conserved residues are drawn in red boxes, similar residues in red type. The secondary structure assignment for Erns is shown at the top. Cysteines involved in intra-chain disulfide bridges are marked by a green number and residues described in the text are marked by a star.

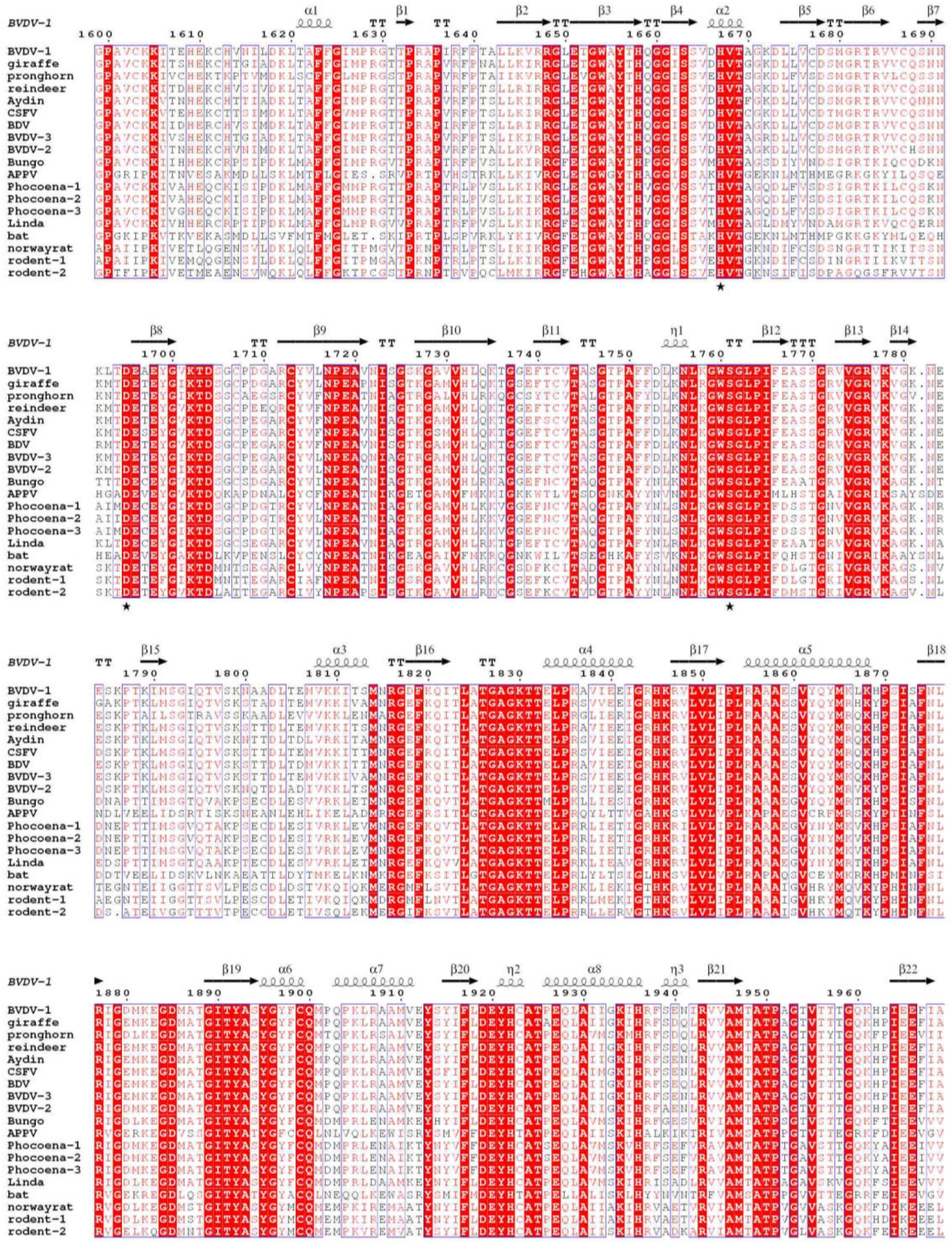
BVDV-1
BVDV-1
giraffe
pronghorn
reindeer
Aydin
CSFV
BDV
BVDV-3
BVDV-2
Bungo
APPV
Phocoena-1
Phocoena-2
Phocoena-3
Linda
bat
norwayrat
rodent-1
rodent-2

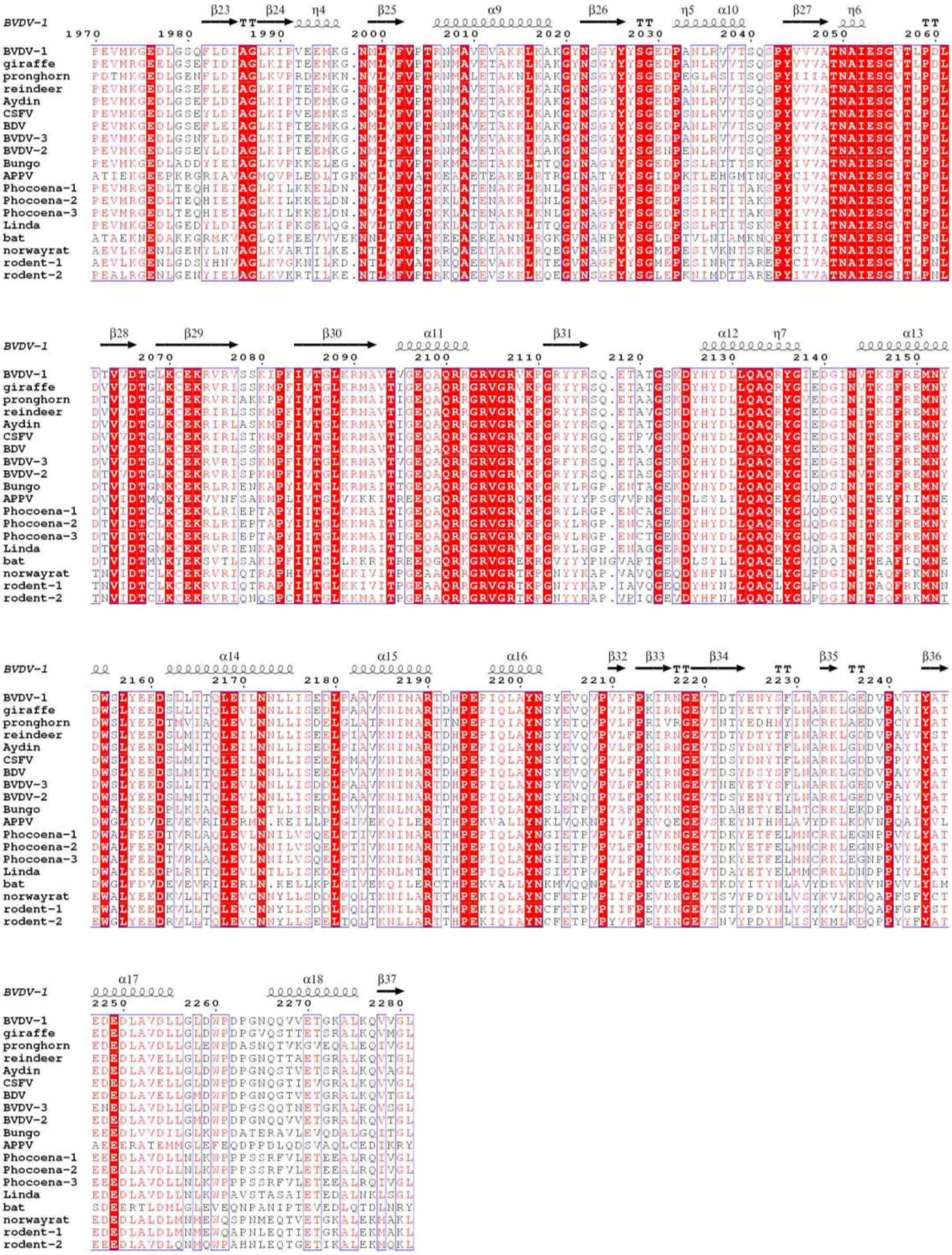

Figure S4. Protein sequence alignment of pestiviruses NS3 proteins. Sequence alignment of BVDV1 NS3 with NS3 proteins from other pestiviruses. Conserved residues are drawn in red boxes, similar residues in red type. The secondary structure assignment for NS3 is shown at the top. Cysteines involved in intra-chain disulfide bridges are marked by a green number and residues described in the text are marked by a star.

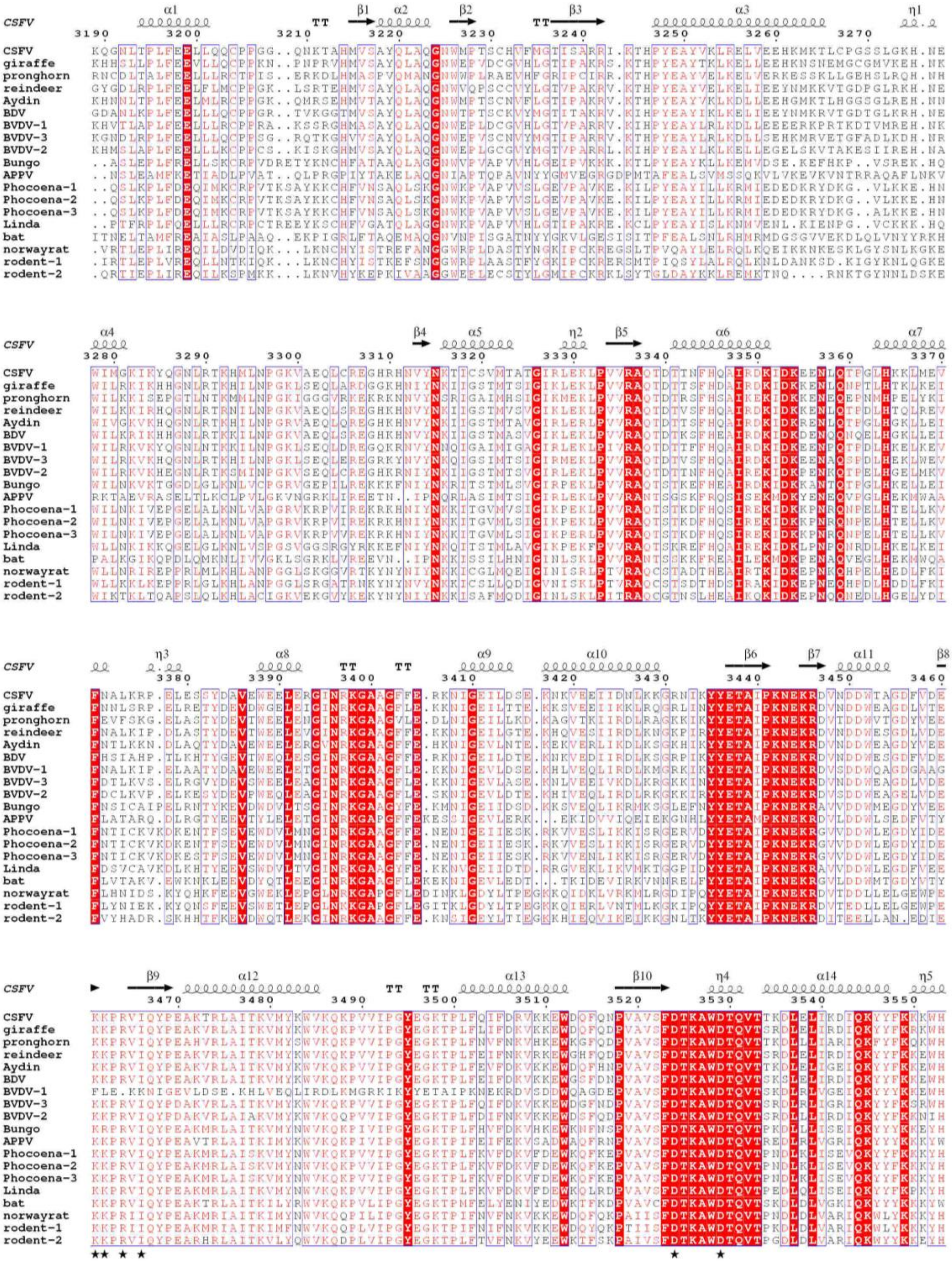
CSFV
CSFV
giraffe
pronghorn
reindeer
Aydin
BDV
BVDV-1
BVDV-3
BVDV-2
Bungo
APPV
Phocoena-1
Phocoena-2
Phocoena-3
Linda
bat
norwayrat
rodent-1
rodent-2

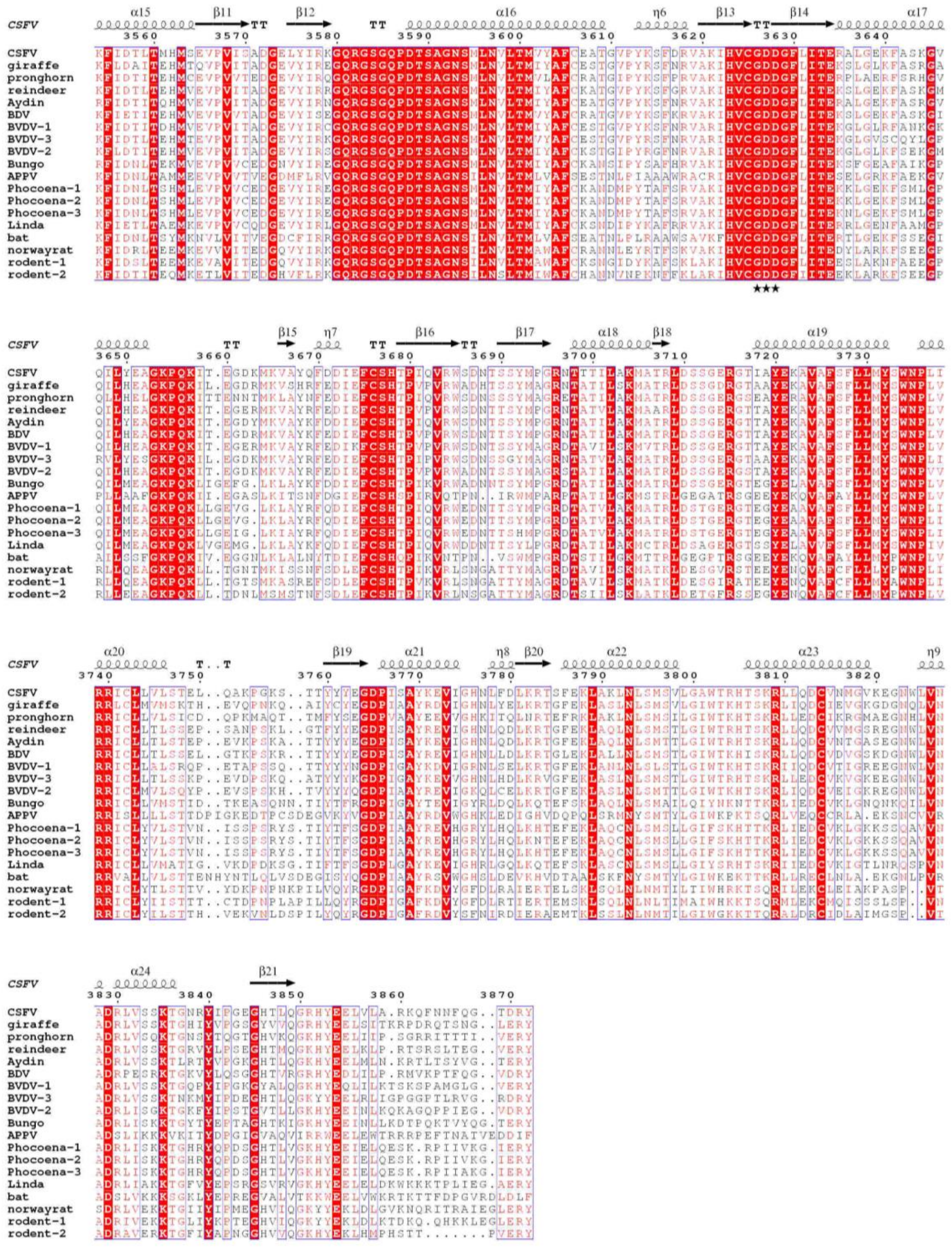

Figure S5. Protein sequence alignment of pestivirus NS5B proteins. Sequence alignment of CSFV NS5B with NS5B proteins from other pestiviruses. Conserved residues are drawn in red boxes, similar residues in red type. The secondary structure assignment for NS5B is shown at the top. Cysteines involved in intra-chain disulfide bridges are marked by a green number and residues described in the text are marked by a star.
